# Proanthocyanidins Attenuation of H_2_O_2_-Induced Oxidative Damage in Tendon-Derived Stem Cells via Upregulating Nrf-2 Signaling Pathway

**DOI:** 10.1155/2017/7529104

**Published:** 2017-10-22

**Authors:** Wenshuang Sun, Jia Meng, Zhenheng Wang, Tao Yuan, Hong Qian, Wenxiang Chen, Jian Tong, Yu Xie, Ya Zhang, Jianning Zhao, Nirong Bao

**Affiliations:** Department of Orthopedics, Nanjing Jinling Hospital, No. 305 Zhongshan East Road, Nanjing 210000, China

## Abstract

Proanthocyanidins (PCs) have shown inhibition of oxidative damage by improving Nrf-2 expression in many tissues. However, the cytoprotective effects of PCs on H_2_O_2_-induced tendon damage have not been verified. The current study was aimed at assessing the cytoprotection of PCs on the oxidative cellular toxicity of tendon-derived stem cells (TDSCs) induced by H_2_O_2_. The TDSCs were isolated from patellar tendons of Sprague Dawley (SD) rats, and the cells after third passage were used for subsequent experiments. The isolated cells were identified by flow cytometry assay and multidifferentiation potential assay. Cell Counting Kit-8 assay was performed to examine cell viability. Real-Time PCR and Western Blot were employed to, respectively, assess the mRNA and protein expressions of Nrf-2, GCLM, NQO-1, and HO-1. PCs significantly improved the cell viability of TDSCs. Furthermore, H_2_O_2_ upregulated Nrf-2, GCLM, NQO-1, and HO-1 without significant difference, while the proteins expressions were increased with significant difference in PCs group and PCs + H_2_O_2_ cotreated group. All the findings indicated that PCs could protect against the oxidative damage induced by H_2_O_2_ in TDSCs, and the cytoprotective effects might be due to the ability of PCs to activate the expressions of GCLM, HO-1, and NQO-1 via upregulating Nrf-2 signaling pathway.

## 1. Introduction

Tendon injuries are some of the most intractable orthopedic problems. Numberous tendon injuries are chronic and degenerative, which result in the formation of fibrovascular scar that never attain the gross, histological, or mechanical characteristics of normal tendon, as tendon has very little regenerative capacity of its own [1]. In the degenerative process, mitochondrial dysfunction causes an overproduction of reactive oxygen species (ROS) [2], which would result in the oxidative damage. The oxidative stress may play a role in tendon degeneration process, with a constant loss of tendon function [3]. This could be more pronounced in the elderly population in whom age-related physiological dysfunctions could impair antioxidant defenses and increase susceptibility to oxidative stress and tendon damage [4]. Therefore, the oxidative damage is inevitable in tendon degeneration, and the theory of oxidative-stress-related tendon damage has attracted increasing attention.

Nowadays, no effective therapies for tendon injury are available, but cell based therapies are fully addressed [5]. An amount of evidence has demonstrated that tendon-derived stem cells (TDSCs) are multipotent [6, 7] and play a major role in the maintenance of tendon homeostasis and recovery after injury [8]. Some studies have indicated that TDSCs effectively promote tendon remodeling in animals tendon injury model [9, 10], and they would be better repaired if they were cotreated with platelet-rich plasma [11], BMP-2 (bone morphogenetic protein-2) [12] or ADSCs (Adipose-Derived Stem Cells) [13], and BMSCs (Bone Marrow Mesenchymal Stem Cells) [14]. However, in the process of tendon degeneration, the oxidative damage to TDSCs could not be avoided either, so we brought TDSCs into our cytological study for determining the effect of oxidative damage on TDSCs, in consideration of the potential of TDSCs in tendon repair.

Proanthocyanidins (PCs) are oligomers and polymers of flavan-3-ols which contain various amounts of catechin and epicatechin [15], and are especially extracted from grape seeds [16]. PCs possess a variety of biological activities, including antioxidant, antitumor, anti-inflammatory, antiallergic, and antitoxic effects [17]. PCs are also powerful natural antioxidants and efficient free radical scavengers, whose antioxidative ability immensely exceeds vitamins C [18]. Cumulative evidence suggests that PCs possess a biological property against oxidative stress in different organs and tissues. A previous study has revealed that ginkgo PCs can effectively lessen cerebral ischemia/reperfusion injury and protect ischemic brain tissue, and these effects are associated with antioxidant properties [19]. Han et al.'s research indicated that PCs with half-dose colistin were equivalent to antibiotic treatment and assisted weaned animals in resisting intestinal oxidative stress by increasing diversity and improving balance of gut microbes [20]. Moreover, one study has reported that grape seed PCs (GSP) might potentially prevent hypoxic pulmonary hypertension via antioxidant and antiproliferative mechanisms [21]. Another study has proved that PCs could enhance the ability of liver tissue to protect against oxidative stress via the Nrf2/ARE signaling pathway, resulting in decreasing ER stress and apoptosis of liver tissue [22]. Also, PCs preadministration could activate the expression of Nrf-2 and decrease the NF-*κ*B activities, suggesting its inhibitory action in inflammatory response by utilizing NF-*κ*B-dependent pathway. PCs induce the activation of PI3K/Akt pathway which regulates the level of Nrf2-dependent inducible expression of HO-1, Trx, and peroxiredoxin I (PrxI) [17].

However, the protective effects of PCs against oxidative damage have not been clarified in TDSCs, and there is no similar study available. So, this study was undertaken to demonstrate the protection against oxidative damage in TDSCs to check whether PCs would, in part, ameliorate the toxic effect of H_2_O_2_. If PCs show protective effects on TDSCs, dietary PCs would serve as medicine against oxidative damage.

## 2. Material and Methods

### 2.1. Isolation and Culture of Rat TDSCs

TDSCs were isolated from the patellar tendon of 8-week-old SD rats. All experiments were approved by the Animal Care and Use Committee of Jinling Hospital. A total of four SD rats, weighting about 250 g, were sacrificed by chloral hydrate overdose. The whole patellar tendons were excised from both limbs of the four SD rats. After careful removal of the peritendinous connective tissue, the samples were stored in PBS (sh30256.01B, Hyclone, USA). The tissues were then minced (1 mm^3^); 100 mg of tissue sample was digested with 3 mg type I collagenase (Sigma, USA) for 2 h at 37°C and passed through a 70 *μ*m cell strainer (Biologix, USA) to yield single-cell suspension. The released cells were centrifuged at 1000 rpm for 5 min and resuspended in growth medium consisting of DMEM/F12 (Hyclone, USA) supplemented with 10% FBS (Hyclone, USA), 100 U/ml penicillin, and 100 *μ*g/ml streptomycin (SV30010, Hyclone, USA). The isolated cells were cultured in T25 flasks at 37°C with 5% CO_2_. After being incubated for 24 h, nonadherent cells were removed by washing with PBS. After a week, the cells were trypsinized as passage 0 (P0). Cells after P3 were used for subsequent experiments.

### 2.2. Identification of Stem Cells with Lineage-Specific Markers

The isolated cells were identified by flow cytometry assay with lineage-specific markers, including anti-CD90 (ab33694, Abcam, UK) and anti-CD31 (ab33858; Abcam, UK). Isotype controls (ab91357, ab91356; Abcam, UK) were used for anti-CD90 and anti-CD31, respectively. TDSCs (5 × 10^5^) at P3 were incubated with fluorescein-conjugated anti-rat monoclonal antibodies for 1 h at 4°C and centrifuged at 1000 rpm for 5 min. The stained cells were resuspended in 500 *μ*L ice-cold PBS containing 10% FBS and analyzed by FACs (FACSCalibur, Becton Dickinson) [23].

### 2.3. Multidifferentiation Potential

The osteogenic, adipogenic, and chondrogenic differentiation potential of TDSCs at P3 were investigated according to Rui et al. [23].

#### 2.3.1. Osteogenic Differentiation Assays

TDSCs were seeded at a density of 4 × 10^4^ cells/well in a 6-well plate and cultured until the cells reached confluence. They were then incubated in complete medium, supplemented with 1 nM dexamethasone, 50 mM ascorbic acid, and 20 mM *β*-glycerol phosphate (Sigma-Aldrich) for 3 weeks. For alizarin red staining, the cells were fixed in 70% ethanol for 10 min and stained with 0.5% alizarin red (Sigma-Aldrich) for 30 min.

#### 2.3.2. Adipogenic Differentiation Assays

TDSCs were seeded with the same density as that indicated for the osteogenic assays. The medium was replaced with complete medium, supplemented with 500 nM dexamethasone, 0.5 mM isobutylmethylxanthine, 50 mM indomethacin, and 10 ug/mL insulin (Sigma-Aldrich). The cells were cultured for 3 weeks for the presence of oil droplets by oil red-O staining. The presence of oil droplets was confirmed by staining the cells with 0.3% fresh oil red-O solution (Sigma-Aldrich) for 2 h after fixation with 70% ethanol for 10 min.

#### 2.3.3. Chondrogenic Differentiation Assays

For chondrogenic differentiation, a pellet culture system was used. About 8 × 10^5^ cells were centrifuged into a pellet at 450*g* for 10 min in a 15 mL tube and cultured in chondrogenic medium, which contained low-glucose Dulbecco's modified Eagle's medium (Gibco, Invitrogen Carlsbad, CA), supplemented with 10 ng/mL transforming growth factor-*β*3 (R&D Systems), 500 ng/mL bone morphogenetic protein-2 (R&D Systems), 10^−7 ^M dexamethasone, 50 ug/mL ascorbate-2-phosphate, 40 ug/mL proline, 100 ug/mL pyruvate (Sigma-Aldrich), and 1 : 100 diluted ITS + Premix (6.25 mg/mL insulin, 6.25 mg/mL transferrin, 6.25 mg/mL selenous acid, 1.25 mg/mL bovine serum albumin, and 5.35 mg/mL linoleic acid) (Becton Dickinson, Franklin Lakes, NJ). After 3 weeks, the pellet was fixed for staining of Alcian blue.

### 2.4. Cell Viability Assessment by the CCK-8 Assay

After trypsinization, P3 TDSCs were seeded at a density of 1 × 10^5^ cells/well in 96-well plates with 100 *μ*L complete medium and incubated for 24 h for subsequent experiments.

#### 2.4.1. PCs and H_2_O_2_ Cytotoxicity Analysis

Seeded TDSCs were treated with different concentrations of PCs and H_2_O_2_ (PCs, 10–130 *μ*g/mL; H_2_O_2_, 200–500 umol/mL) for 2 h to assess cytotoxic effects. Treatment with 10 *μ*L CCK-8 (C0038, Biyuntian, China) per well for 2 h was performed. Conversion of WST to formazan was measured at 450 nm on a microplate spectrophotometer (51119200, Thermo Scientific Multiskan, USA). Cytotoxicity assay for PCs was to rule out their cytotoxic effects on TDSCs, while that of H_2_O_2_ was to assess the IC_50_ value for subsequent studies.

#### 2.4.2. Antioxidant Test of PCs

The cytoprotective effects of PCs on H_2_O_2_-induced oxidative stress were determined. TDSCs were pretreated with PCs at concentrations of 0, 10, 50, and 90 *μ*g/mL for 2 h followed by H_2_O_2_ treatment for 2 h. Treatment groups for subsequent experiments were as follows: group I, control; group II, H_2_O_2_ (at IC_50_); group III, PCs (10 *μ*g/mL) + H_2_O_2_ (IC_50_); group IV, PCs (50 *μ*g/mL) + H_2_O_2_ (IC_50_); group V, PCs (90 *μ*g/mL) + H_2_O_2_ (IC_50_).

### 2.5. Real-Time PCR

Gene expression in TDSCs was assessed by Real-Time PCR. TDSCs were seeded in 6-well plates at a density of 1 × 10^5^ in growth medium. After 24 h, the cells were treated with 100 *μ*g/mL PCs or 200 *μ*mol/mL H_2_O_2_ or treated with both one by one. After incubation for 24 h, total RNA was extracted from the TDSCs using RNA Extraction Kit (No. 9767, TAKARA, Japan). First-strand cDNA was synthesized in a 10 *μ*L reaction from 500 ng total RNA by reverse transcription with PrimeScript RT Master Mix (number RR036A, TAKARA, Japan). The Real-Time PCR program for cDNA synthesis was as follows: 37°C for 15 min followed by 85°C for 5 s, with a hold at 4°C. RT-PCR was carried out by using SYBR Premix Ex Taq II (TIi RNaseH Plus) (number RR820A, TAKARA, Japan). The assessed genes were Nrf-2 (nuclear factor erythroid-derived factor 2-related factor), GCLM (glutamate-cysteine ligase regulatory subunit), HO-1 (hemoxygenase-1), NQO-1 (NADPH: quinone oxidoreductase), and GAPDH (used as an internal control). Forward and reverse primers were synthesized by GenePharma (Shanghai, China) and listed in Table 1. After initial denaturalization for 30 s at 95°C, PCR was performed for 40 cycles of denaturalization for 5 s at 95°C and annealing for 34 s at 60°C. At least three independent experiments were performed to obtain the relative expression levels for each gene.

### 2.6. Western Blot

TDSCs were treated with H_2_O_2_ or pretreated with PCs for 24 h as for Real-Time PCR. Afterward, TDSCs were washed with PBS, lysed in lysis buffer, and kept on ice for 5 min by Whole Protein Extract Kit (Jiancheng Bioengineering, Nanjing). Cell lysates were centrifuged at 20,000 rpm at 4°C for 5 min, and the supernatants were stored at −80°C until use. Protein concentrations were measured by using a protein assay kit (Jiancheng Bioengineering, Nanjing). Twenty micrograms of total protein were diluted in loading buffer, separated by SDS/PAGE, and electroblotted onto PVDF membranes. The membranes were then blocked with TBS-Tween 20 solution containing 5% nonfat dry milk and incubated overnight at 4°C with specific antibodies against Nrf-2 (1 : 200) (ab31163, Abcam, USA), GCLM (1 : 1000) (ab126704, Abcam, USA), HO-1 (1 : 200) (ab68477, Abcam, USA), and *β*-actin (ab8226, Abcam, USA). Proteins were visualized using goat anti-rabbit conjugated to HRP and a Chemiluminescence Western Blotting Detection system (34079, Thermo Pierce, USA). Protein band intensities were quantified using the Quantity One software.

### 2.7. Statistical Analysis

All calculations and statistical analyses were performed with SPSS (V19.0) and GraphPad (V6.0). Values were expressed as mean ± SD and analyzed by one-way analysis of variance (ANOVA). *p* < 0.05 was considered statistically significant.

## 3. Results

### 3.1. Isolation and Identification of TDSCs

#### 3.1.1. Cell Morphology of TDSCs at Different Passages

**Figure 1:**
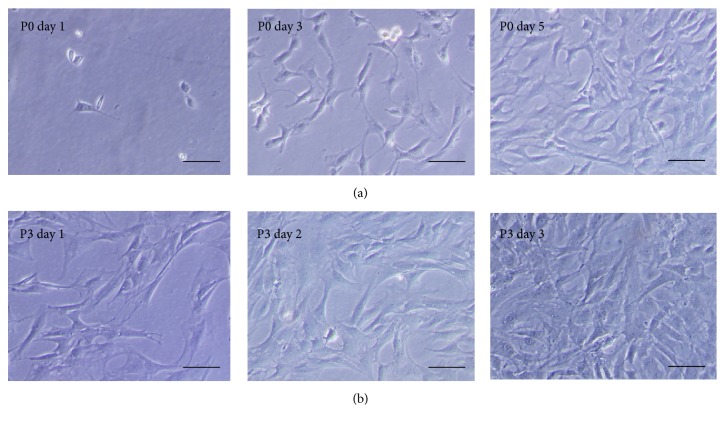
TDSCs culture and characterization. Photomicrographs showed the TDSCs morphology at different passages. (a) At P0, TDSCs looked like large polygonal and star-shaped cells. (b) At P3, a homogeneous population of fibroblast-like cells was obtained. Magnification: ×100. Cell size bar: 100 um.

TDSCs were heterogeneous in size and density, while reflecting some differences in cell morphology and proliferation. At P0, TDSCs looked like large polygonal and star-shaped cells. At P3, a homogeneous population of fibroblast-like cells was obtained. TDSCs proliferated slowly at P0; however, they grew much faster at P3 and overspread the cell culture flask at day 3 (Figure 1).

#### 3.1.2. Flow Cytometric Analysis of MSC Markers

**Figure 2:**
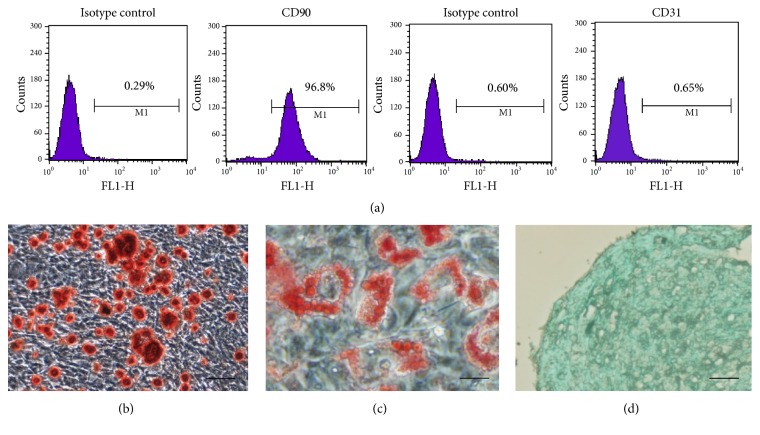
The isolated cells were identified by flow cytometry assay with lineage-specific markers and multidifferentiation potential assays. (a) Graphs showed the expression levels of the mesenchymal stem (CD90) and endothelial (CD31) cell markers on TDSCs. Compared to the isotype control, a high percentage of cells expressed CD90, whereas only few cells showed CD31. (b) Osteogenic differentiation assays. Alizarin-red-positive calcium nodules were observed after osteogenic induction of the cells for 3 weeks. Magnification: ×100. Cell size bar: 100 um. (c) Adipogenic differentiation assays. Lipid droplets were formed after incubating the cells in complete medium with adipogenic supplements for 3 weeks. Magnification: ×400. Cell size bar: 25 um. (d) Chondrogenic differentiation assays. Cartilage-like tissues with Alcian blue stained acid glycosaminoglycan were observed, which indicated the formation of extracellular cartilage matrix. Magnification: ×100. Cell size bar: 100 um.

To confirm that the obtained TDSCs were stem cells, the expression levels of MSC surface markers were determined, including CD90 and CD31, by flow cytometric analysis. The results showed that 96.8% of the TDSCs were positive for the fibroblastic marker CD90 and negative for the endothelial cell marker CD31, as compared to the isotype controls (Figure 2(a)).

#### 3.1.3. Multidifferentiation Potential of TDSCs Colonies

*Osteogenic Differentiation Assays*. The osteogenic differentiation potential of the TDSCs colonies was determined and alizarin-red-positive calcium nodules were observed after osteogenic induction of the cells for 3 weeks (Figure 2(b)).

*Adipogenic Differentiation Assays*. Lipid droplets were formed after incubating the cells in complete medium with adipogenic supplements for 3 weeks (Figure 2(c)).

*Chondrogenic Differentiation Assays*. After chondrogenic induction of TDSCs for 3 weeks, cartilage-like tissues with Alcian blue stained acid glycosaminoglycan were observed, which indicated the formation of extracellular cartilage matrix (Figure 2(d)).

According to cell morphology, flow cytometric analysis of MSC markers, and multidifferentiation potential assays, we confirmed that the isolated cells were TDSCs, ruling out contamination by other cells.

### 3.2. The Cytotoxic Effects of PCs and H_2_O_2_ on TDSCs

**Figure 3:**
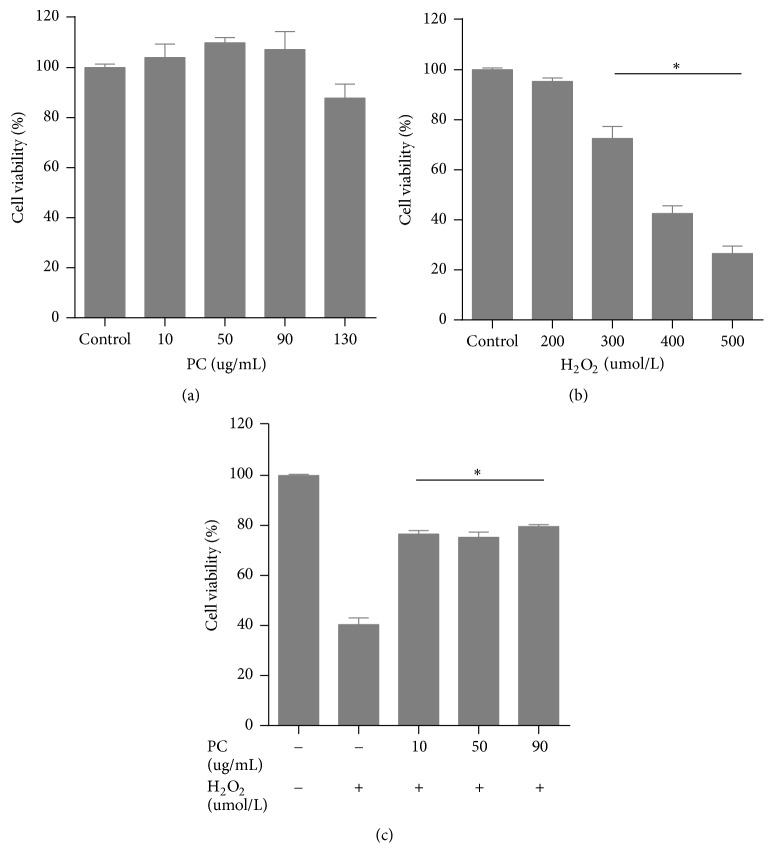
PCs reduced H_2_O_2_-induced oxidative stress in TDSCs. (a) PCs had no cytotoxic effects on TDSCs (*p* > 0.05). (b) H_2_O_2_ reduced cell viability (%) in TDSCs; data are percentage of cell viability in comparison to the control group, and viability in the 400 *μ*mol/ml was approximately 50%, reflecting IC50. (c) PCs protected TDSCs against H_2_O_2_-induced oxidative stress. Obvious differences were observed between the non-PCs and pre-PCs treatment groups, but no significant differences were found among groups pretreated with PCs at various concentrations. H_2_O_2_ (+): 400 *μ*mol/ml. All results were expressed as the mean ± SD (*n* = 3); ^*∗*^*p* < 0.05, as compared to control group.

Before using PCs and H_2_O_2_ for TDSCs treatment, we assessed their cytotoxicity to rule out PCs cytotoxicity and determine the IC_50_ value of H_2_O_2_. All PCs amounts showed no cytotoxicity on TDSCs (*p* > 0.05). Meanwhile, the cells treated with H_2_O_2_ at high concentrations were overtly inhibited; the cell viability (%) obtained with 400 *μ*mol/ml was approximately 50%, and this concentration was considered the IC_50_ for subsequent experiments. Cytoprotection was achieved by pretreatment with PCs (*p* < 0.01), with a maximum (*p* < 0.001) at 90 *μ*g/ml. For the convenience of sample preparation, 100 *μ*g/ml pretreatment was used for determining oxidative stress caused by H_2_O_2_ (Figure 3).

### 3.3. The Effects of PCs on H_2_O_2_-Induced Alteration of Nrf-2, GCLM, HO-1, and NQO-1 Expression Levels

**Figure 4:**
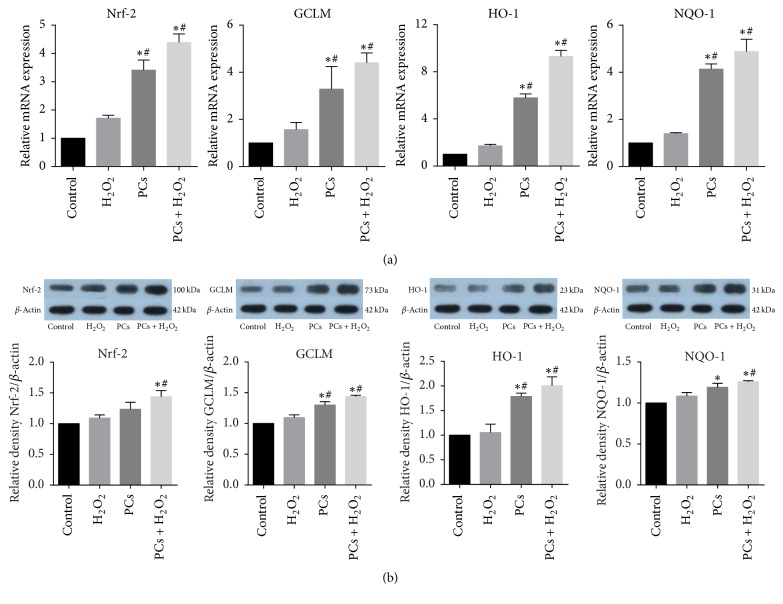
The effects of PCs on H_2_O_2_-induced alterations of Nrf-2, GCLM, HO-1, and NQO-1 mRNA and protein levels. (a) The effects of PCs on alterations of mRNA levels. (b) The effects of PCs on alterations of protein levels. All results were expressed as the mean ± SD (*n* = 3); ^*∗*^*p* < 0.05, as compared to control group; ^#^*p* < 0.05, as compared to H_2_O_2_ group.

To evaluate whether Nrf-2 activation played a role in PCs protection against the oxidative damage induced by H_2_O_2_, the expression of Nrf-2 and its downstream genes including GCLM, HO-1, and NQO-1 in TDSCs was measured. As shown in Figure 4(a), the Real-Time PCR results showed that mRNA expression levels of Nrf-2 and its downstream genes in the H_2_O_2_ group were increased without significant difference (*p* > 0.05), while expressions of these genes were increased with significant difference in the PCs group and PCs/H_2_O_2_ cotreated group (*p* < 0.05) compared with the control group. The expressions of Nrf-2 and its downstream genes in the PCs group and PCs/H_2_O_2_ cotreated group were significantly increased (*p* < 0.05) compared with the H_2_O_2_ group. Meanwhile, as shown in Figure 4(b), the Western Blot results presented the same feature (*p* < 0.05), except Nrf-2 and NQO-1 in PCs group (*p* > 0.05).

## 4. Discussion

The current study demonstrated that PCs had cytoprotective effects on oxidative cytotoxicity to TDSCs. These effects were achieved via upregulating Nrf-2 signaling pathway. Our results indicated that the oxidative stress induced by H_2_O_2_ indeed caused the oxidative damage, and the efficient antioxidant effects of PCs were also available in TDSCs.

PCs are extremely efficient natural antioxidants, their antioxidant activity is 20 times higher than that of vitamin C, and the antioxidant effects have been revealed in a range of studies. According to the cytotoxicity analyses by CCK-8 assay, it was shown that PCs had no cytotoxic effects on TDSCs, and they did not influence the cell viability of TDSCs. The cell viability of TDSCs treated with H_2_O_2_ was obviously decreased, resulting from the H_2_O_2_-induced oxidative damage. However, the cell viability of TDSCs pretreated with PCs was distinctly improved, even at a very low concentration, and this exhibited the efficient antioxidant effects of PCs on TDSCs.

Currently, Nrf-2 is the key molecule which mediates the response of the endogenous antioxidant system. Under basal homoeostatic redox conditions, Nrf-2 is blocked in the cytoplasm by Keap-1 via one high-affinity site and one low-affinity binding site [24]. In response to both oxidative and electrophilic stressors, the Keap-1/Nrf-2 interaction is disrupted, allowing Nrf-2 to translocate to the nucleus and activate downstream genes. Previous studies have suggested that Nrf-2 plays a crucial role in cellular resistance to oxidative and exogenous damage [25, 26], and activation of Nrf-2 can improve the expression of antioxidant genes and induce synthesis of phase II detoxifying enzymes [27]. The Nrf-2/ARE pathway is also capable of stimulating the activity of superoxide dismutase (SOD), hemoxygenase-1 (HO-1), NADPH: quinone oxidoreductase (NQO1), and glutamate-cysteine ligase regulatory subunit (GCLM) [28].

In our study, the results showed that the gene transcription level of Nrf-2 was increased in the H_2_O_2_ group. This result indicated that, in order to cope with the oxidative damage induced by H_2_O_2_, the TDSCs activated Nrf-2 signaling pathway to compensate for the oxidative damage [25–27]. When the TDSCs were treated with PCs, the Nrf-2 mRNA and protein expressions were also elevated with a significant difference compared with control group and H_2_O_2_ group. This result indicated that PCs could activate the expression of Nrf-2, and the protective effect was related to Nrf-2 signaling pathway. Previous studies have demonstrated that the activation of Nrf-2 could improve the expression of antioxidant genes, including GCLM, NQO-1, and HO-1 [28]. Our results showed that PCs could upregulate Nrf-2 downstream gene GCLM, NQO-1, and HO-1 expression, resulting in enhancing the ability of TDSCs to resist the oxidative damage induced by H_2_O_2_. Combined with our previous results, the cell viability was significantly improved in the group cotreated with PCs, resulting from the upregulation of these genes via Nrf-2 signaling pathway. Although our study had proved the efficient antioxidant effects of PCs on TDSCs, the molecular mechanism responsible for the activation of Nrf-2 was not clarified and was worthy of further investigation.

The study had provided evidence that PCs had efficient antioxidant effects on TDSCs, and our results suggested that PCs protected TDSCs against oxidative damage via Nrf-2 signaling pathway. These findings may be attributed to the manifold effects of PCs as functional foods in future application.

## 5. Conclusion

In conclusion, our study indicated that PCs could protect against the oxidative damage induced by H_2_O_2_ in TDSCs, and the cytoprotective effects might be achieved by the fact that PCs activated the expression of GCLM, HO-1, and NQO-1 via upregulating Nrf-2 signaling pathway.

## Figures and Tables

**Table 1:** Primers for Real-Time PCR.

Gene	Type	Primers	Size
Nrf-2	F	5′-GGACATGGAGCAAGTTTGGC-3′	102
	R	5′-GGGCTGGGGACAGTGGTAGT-3′	
GCLM	F	5′-ATCATGGCTTCCCCTCCAAT-3′	70
	R	5′-CCTCCCAGTAAGGCTGCAAAT-3′	
NQO-1	F	5′-CGGTGAGAAGAGCCCTGAT-3′	111
	R	5′-CGACCACCTCCCATCCTT-3′	
HO-1	F	5′-TCACCTTCCCGAGCATCGA-3′	119
	R	5′-GGCGGTCTTAGCCTCTTCTGT-3′	
GAPDH	F	5′-AGGTCGGTGTGAACGGATTTG-3′	95
	R	5′-GGGGTCGTTGATGGCAACA-3′	
